# Nanomedicine in Hepatocellular Carcinoma: A New Frontier in Targeted Cancer Treatment

**DOI:** 10.3390/pharmaceutics14010041

**Published:** 2021-12-25

**Authors:** Anita Bakrania, Gang Zheng, Mamatha Bhat

**Affiliations:** 1 Toronto General Hospital Research Institute, Toronto, ON M5G 2C4, Canada; Anita.Bakrania@uhnresearch.ca; 2 Ajmera Transplant Program, University Health Network, Toronto, ON M5G 2N2, Canada; 3 Princess Margaret Cancer Centre, University Health Network, Toronto, ON M5G 2C1, Canada; gang.zheng@uhnresearch.ca; 4 Institute of Biomedical Engineering, University of Toronto, Toronto, ON M5S 3G9, Canada; 5 Department of Medical Biophysics, University of Toronto, Toronto, ON M5G 1L7, Canada; 6 Division of Gastroenterology, Department of Medicine, University Health Network, Toronto, ON M5G 2C4, Canada; 7 Department of Medical Sciences, University of Toronto, Toronto, ON M5S 1A1, Canada

**Keywords:** hepatocellular carcinoma, liver cancer, nanoparticle, nanomedicine, gene therapy

## Abstract

Hepatocellular carcinoma (HCC) is the third leading cause of cancer-related death and is associated with a dismal median survival of 2–9 months. The fundamental limitations and ineffectiveness of current HCC treatments have led to the development of a vast range of nanotechnologies with the goal of improving the safety and efficacy of treatment for HCC. Although remarkable success has been achieved in nanomedicine research, there are unique considerations such as molecular heterogeneity and concomitant liver dysfunction that complicate the translation of nanotheranostics in HCC. This review highlights the progress, challenges, and targeting opportunities in HCC nanomedicine based on the growing literature in recent years.

## 1. Introduction

Hepatocellular carcinoma (HCC) is the predominant form of primary liver cancer and the fifth most common cancer globally [1]. It has a high mortality rate causing over 600,000 deaths annually worldwide. Due to the insidious growth nature of HCC, the majority of patients are diagnosed at advanced stages of the disease, at which point available therapeutic options are limited and ineffective [2].

Cirrhosis arises in the setting of chronic liver diseases such as hepatitis C (HCV) infection, alcoholic liver disease (ALD), non-alcoholic steatohepatitis (NASH), and hemochromatosis (resulting in liver iron overload). HCC does also arise in the setting of hepatitis B virus (HBV) infection even without cirrhosis, given the ability of HBV to stimulate oncogenesis through integration into the liver cell DNA [3]. Additionally, aflatoxin B1, a secondary metabolite of a few Aspergillus fungi species, is a known potent mycotoxin ingested through staple food contamination and a major cause of HCC in high-risk geographical regions of Africa and Asia [4,5,6]. Furthermore, anti-diabetic agents such as sulfonylureas and insulin [7,8] are associated with an increased risk of HCC in contrast to metformin [7,9,10,11,12] and thiazolidinediones [7,13], which are associated with a reduced risk of HCC development. Other medications such as statins [14,15] and aspirin [16] have been shown to reduce the risk of HCC incidence [17].

The treatment of HCC depends on various factors, including the tumor stage, patient performance status, and liver functional reserve, thereby requiring a multidisciplinary approach. Current treatment options reserved for early-stage HCC include local ablative therapies, resection, and liver transplantation [18]. Systemic therapies and transarterial radioembolization (TACE) are the only available treatment options for advanced-stage disease [19,20]. Among the targeted therapies, sorafenib was the first systemic drug that showed efficacy in advanced HCC and had remained the standard of care as first-line therapy for over 10 years [21,22,23,24]. After a decade of negative trials, the approvals of lenvatinib as first-line along with regorafenib, carbozantinib, and recombinant immunoglobulin G1 (IgG1) monoclonal antibody ramucirumab as second-line post-sorafenib in early 2019 paved the way to sequential systemic therapy in HCC [25,26,27,28,29,30]. Recently in 2020, the combination of atezolizumab and bevacizumab also received FDA approval and showed significant improvement in the overall and progression-free survival outcomes compared to sorafenib in unresectable or metastatic HCC [31,32]. Atezolizumab selectively targets programmed death ligand 1 (PD-L1), thus reversing T-cell suppression, while bevacizumab is a monoclonal antibody targeting vascular endothelial growth factor (VEGF), thus inhibiting angiogenesis and tumor growth [33,34,35].

Alongside the glimmers of real hope with the enormous recent progress in HCC therapy and opportunities that lie ahead, the current challenges cannot be overlooked. The current overall outcome still remains unsatisfactory, with median survivals in early and advanced HCC of 6–9 months and 1–2 months, respectively [36]. The mortality is often not due to the tumor itself but the complications associated with cirrhosis, such as ascites, variceal hemorrhage, hepatic encephalopathy, and hepatorenal syndrome. Henceforth, therapeutic strategies should aim at not only eradicating tumors but also, more specifically, targeting the tumor cells and strengthening the host immune system. Additionally, these drugs are associated with significant side effects, particularly in patients with concomitant cirrhosis and liver dysfunction.

Chemotherapeutic agents focus on specific carcinogenic pathways to limit systemic side effects. Unfortunately, there is no single dominant pathway that exists for the development of HCC. Overexpression of multiple signaling pathways has been implicated in the pathogenesis of HCC, including epidermal growth factor (EGF), VEGF, Ras mitogen-activated protein kinase (MAPK), insulin-like growth factor receptor (IGFR), phosphoinositide 3-kinase (PI3K)/phosphatase, and tensin homolog (PTEN)/Akt/mammalian target of rapamycin (mTOR), hepatocyte growth factor/c-MET and Wnt/β-Catenin pathways providing a wide scope to hunt newer targets for HCC treatment.

Nanotechnology has emerged as an immensely advancing field and a novel possibility to overcome current challenges in HCC therapy. This is largely attributable to its unique features for drug delivery, specific targeting, enhancement of pharmaceutical properties, co-delivery of multiple drugs, visualization of sites of drug delivery by combination with imaging modalities, and the therapeutic nature of some nanomaterials themselves, for example, gold nanoshells and nanorods [37,38,39,40,41,42,43,44]. However, rapid sequestration of particulate carriers by Kupffer cells severely limits the tumoral hepatocyte accumulation, seriously compromising therapeutic efficacy. Therefore, targeted delivery to tumoral hepatocytes via receptors that are overexpressed on these cells represents a promising strategy for HCC targeting [45]. This review aims to identify gaps in our understanding of HCC targeting and why nanomedicine has yet to fulfill its promise in HCC treatment and to offer an overview of the emerging targeting opportunities in the field of nanotherapeutics for HCC treatment.

## 2. Progress in HCC Nanomedicine

Nanotechnology has been progressively advancing ever since it was first introduced in 1974, with exceptional development in cancer research [46,47]. With the augmenting interest in HCC nanomedicine, several studies have been conducted and are currently ongoing to overcome the challenges in specific targeted drug delivery to HCC. Although most nanoparticles tend to accumulate in the liver, thereby defining it as an easy target, the occurrence of HCC in the setting of cirrhosis makes it a greater challenge than expected. Since most drugs must pass through the liver as the primary metabolism site, the altered pharmacokinetics in a cirrhotic liver represents a challenge. Along with that, selectively targeting tumoral hepatocytes defines another obstacle. Therefore, a selectively targeted therapy is desirable in order to selectively target the affected cells while reducing the toxicity of the therapy [48]. Evolving research has developed a broad range of nanoparticles for HCC, which include alumina NPs [49], arsenite NPs [50,51,52], albumin NPs [53], calcium NPs [54,55,56,57], chitosan NPs [58,59,60,61,62], gold NPs [63,64,65,66,67,68,69,70,71,72,73], halifum oxide NPs [74], iron oxide NPs [75,76,77,78,79,80], lipid NPs [81,82,83,84], poly(ethylene glycol) (PEG) NPs [85,86,87,88,89,90,91,92,93,94], platinum NPs [95,96], poly(lactic-co-glycolic acid) (PLGA) NPs [87,88,89,90,91,92,93,94,97,98,99,100,101,102], polysaccharide NPs [45,103,104], selenium NPs [105,106,107], silica NPs [108,109,110,111,112,113,114,115,116,117,118], silver NPs [119,120,121,122], zinc oxide NPs [123], etc. [47].

Subsequently, there has also been progress in the drug/cargo of interest to be delivered through the above-mentioned nano vehicles, with the most recent strategy being gene engineering [124,125,126,127]. Since the primary cause of HCC tumors lies in the dysregulation of various proto-oncogenes and tumor-suppressive genes of several signaling pathways, genome engineering offers a unique approach to HCC treatment via nanotechnology by introducing nucleic acids that edit and code for the abnormal gene and/or suicide gene through interfering RNAs such as miRNA, siRNA, piRNA, and shRNA [128,129,130,131,132,133,134,135,136,137,138,139,140].

## 3. Current Challenges in Treating HCC

### 3.1. Tumor Microenvironment

Recent research has highlighted the link between tumor cells and their surrounding microenvironment along with the fundamental role of the tumor microenvironment in hepatocarcinogenesis [141]. The tumor microenvironment is composed of; (1) cells such as hepatic stellate cells, fibroblasts, immune cells, including regulatory and cytotoxic T cells and tumor-associated macrophages (TAMs), and endothelial cells, (2) proteolytic enzymes including matrix metalloproteinases (MMPs) and tissue inhibitor of metalloproteinases (TIMPs), (3) growth factors, for example, transforming growth factor b1 (TGF-β1) and platelet-derived growth factor (PDGF), 4) inflammatory cytokines, and (5) extracellular matrix (ECM) [142,143,144,145]. An interesting phenomenon called enhanced permeability and retention (EPR), responsible for the formation of leaky vessels and pores of diameter 100 nm to 2 µm, thereby providing an advantage to the design of novel antitumor nanoparticles for tumor targeting in all types of tumors [146,147,148,149]. The EPR effect increases nanoparticle accumulation at the tumor site resulting in a more specific therapeutic targeting along with reduced toxicity of the therapeutic agents due to membrane hyperpermeability and absence of basement membrane in the tumor vasculature compared to normal tissue blood vessels [150,151,152]. Although the liver vasculature inherently possesses leaky vessels, the vasculature abnormalities in the presence of chronic liver diseases such as cirrhosis are ubiquitous [153]. Therefore, designing and developing different nano systems with particle size within the vasculature to selectively target HCC tumor cells would enable an effective drug delivery system in the setting of liver diseases. Another characteristic of the HCC tumor environment is the low extracellular pH, which lies between 6.0 and 7.0 as compared to normal tissues and blood with pH 7.4. This is due to the increased rate of glycolysis leading to accumulation of lactic acid in hypoxic tumor cells [154,155,156,157]. Changes in pH play a role in the delivery of therapeutic agents to the liver tumor cells: an acidic pH favors the cellular uptake of weakly acidic drugs and delays the uptake of weakly basic drugs. This consideration can also inform the synthesis of nanoparticles to provide optimal HCC tumor targeting.

### 3.2. Physiological Barriers to Nanomedicine Targeting HCC Cells

#### 3.2.1. Coronal Protein Adsorption

Protein corona refers to the bound or adsorbed proteins around nanoparticles while they are exposed to various physiological fluids in the systemic circulation. Some of the most abundant proteins include transferrin, fibrinogen, albumin, complement C3, haptoglobin, α-2-macroglobulin, and immunoglobulins A, M, and G [158]. This exposure is inevitably responsible for altering their overall pharmacological and toxicological profile and ultimately triggers an unpredictable therapeutic function. Some of the physicochemical factors responsible for coronal protein adsorption include nanoparticle material hydrophilicity/hydrophobicity, surface charge, size, and shape [158,159,160,161,162]. Studies have shown that a decrease in nanoparticle size decreases the surface interaction impacting the protein conformation, and similarly, a spherical shape of nanoparticles offers increased mass/surface ratio that overall reduces interaction with the proteins in the environment [163,164,165]. Additionally, the nature of the nanoparticle also plays a crucial role in the formation of the protein corona such that hydrophilic nanoparticles attract charged proteins via electrostatic forces, while hydrophobic nanoparticles bind to hydrophobic proteins through van der Waal’s or π-π interactions [162,166]. Lastly, the surface charge promotes protein corona formation in instances where the nanoparticles are highly charged as compared to slightly negatively charged nanoparticles, which offer the least protein interactions and therefore avoid the corona formation [167,168].

Over the past few years, there has been progressive understanding regarding the effect of protein coronas on nanoparticles. Taking a glance at its formation, initially, when the nanoparticles encounter biological fluids, a “soft” corona is formed that represents a loosely bound and rapidly exchanging layer of the highly abundant proteins adsorbed during circulation. Following this, it is further coated with other proteins of high affinity as per the Vroman effect and is now termed as “hard” corona [169,170,171]. This leads to a decrease in enthalpy and displacement of the hydration layer surrounding the nanoparticles, subsequently enhancing entropy. Eventually, this causes (i) increased nanoparticle solubility in an aqueous environment, (ii) enhanced protein aggregation and misfolding, (iii) protein conformational changes leading to phagocytosis, and (iv) mask the nanoparticle components defining its pharmacological function [161]. Additionally, the HCC environment plays a major role in coronal protein adsorption, including its composition, exposure time, pH, temperature, and shear stress [172,173,174]. These factors have the capacity to influence the composition of the protein corona as well. Several strategies have been employed to date to overcome this phenomenon; for example, nanoparticle preparation with carbohydrate moieties, dysopsonic proteins, zwitterions, and hydrophilic “stealth” polymers such as PEG could help escape opsonization, ensuring efficient delivery of the nanoparticles [175,176,177,178,179]. In HCC, it is important to enhance the targeting capability of nanoparticles without the hindrance of the protein corona for the most efficient and optimal targeted therapy considering the above-mentioned challenges that pre-exist in HCC targeting. This can be achieved by personalizing the protein corona using specific noncovalent antibodies for HCC or new targeting ligands considering disease heterogeneity [180,181,182].

#### 3.2.2. Mononuclear Phagocyte System

The mononuclear phagocyte system (MPS), previously known as the reticulo-endothelial system (RES) of the human liver, is composed of three major types of cells, namely, monocytes, macrophages (Kupffer cells), and dendritic cells that play a vital role in immune response balance through their function in antigen presentation and therefore, rapidly sequester nanoparticles upon entry to the liver [183,184,185,186,187,188]. Although in-depth research is of the utmost importance to accurately define the subsets of the MPS cells within the liver tissue, it has been reported that the resident phagocytosing Kupffer cells of the liver potentially uptake and get rid of systematically circulating nanoparticles based on various factors, including size and charge [189,190]. This leads to significant restriction to HCC targeting, and therefore, it is vital for nanoparticles to escape this non-specific uptake by Kupffer cells before they reach the HCC cells. The effect of removing these live phagocytosing cells during nano delivery has been investigated. The results showed that although there was an improvement in tumor targeting, nanoparticle delivery efficiency was not drastically changed, and these cells contributed merely 2%, which concludes that designing nanoformulations should be a balancing act between tumor cell specificity, organ targeting, and escape from phagocytosis [191,192,193]. The most common technique used to overcome phagocytosis by the liver involves the modification of nanoparticle surfaces with charge-neutral, highly hydrophilic, “stealth” polymers such as PEG and poly(vinyl alcohol) (PVA) [194,195]. Another approach is to apply zwitterionic coatings on nanoparticles that are overall neutral in charge but dually contain positively and negatively charged groups such as poly(carboxybetaine), poly(sulfobetaine), 2-methacryloyloxyethyl phosphorylcholine, and poly(maleic anhydride-*alt*-1-alkene) derivatives as a crucial step for more directed delivery of the nanoparticulate system into HCC cells [196].

## 4. Targeting Opportunities for HCC Nanomedicine

### 4.1. Surface Biomarkers for Specific Targeting to HCC

#### 4.1.1. Asialoglycoprotein Receptor (ASGPR)

**Figure 1:**
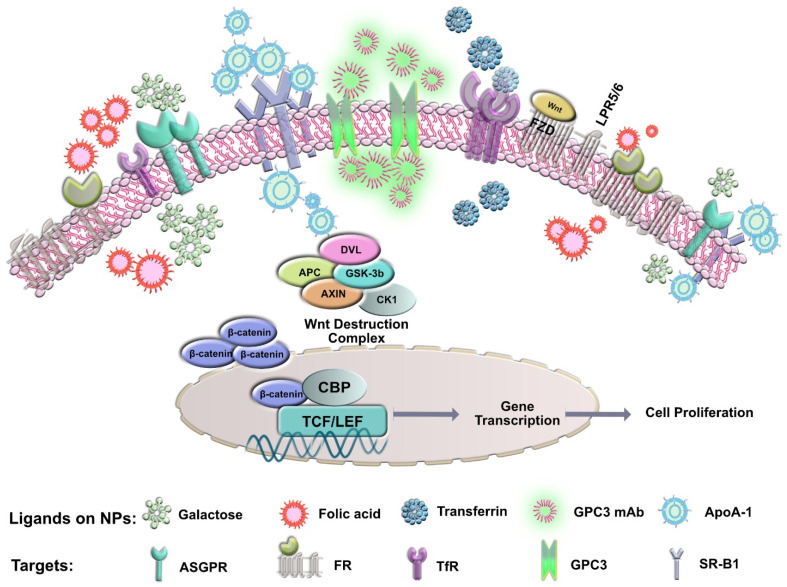
Illustration showing specific targets and ligands for HCC nanomedicine.

ASGPR was first identified by Morell and Ashwell in 1974 as a 40–50 kDa noncovalent hetero-oligomer composed of two homologous poly-peptides with the major and minor subunits HL-1 (hepatic lectin, or ASGPR1, ASGR1) and HL-2 (ASGPR2 or ASGR2), respectively [197,198,199,200]. It is a trans-membrane molecule found in abundance on hepatocytes (500,000 ASGPR/hepatocyte) and specifically expressed on the sinusoidal and basolateral hepatocellular membranes excluding the bile canalicular membrane [201]. The hepatic ASGPR plays an essential role in the clearance of desialylated proteins from the serum via endocytosis and lysosomal degradation. In addition, hepatic ASGPR is involved in the binding, internalization, and degradation of extracellular glycoproteins with exposed terminal galactose, lactose, or N-acetyl-galactosamine residues making it an ideal receptor for galactose-mediated delivery of anti-cancer drugs to the liver (Figure 1) [202,203,204]. The natural ligands of ASGPR include asialoorosomucoid (ASOR, high-affinity ligand with *Ki* = 1.7 nM; 20 Gal), asialoceruloplasmin (86 nM; 12 Gal), asialofectin (17 nM; 12 Gal, 3 GalNAc), and asialotransferrin (3300 nM; 5 Gal) where Gal and GalNAc define the number of galactoses and galactosamines, respectively [205]. Several studies have been conducted to understand the role of targeting ASGPR for HCC after the proof-of-concept that demonstrated that GalNAc-conjugated siRNA specifically accumulated in the hepatocytes [206,207]. Interestingly, ASPGR targeting has also been studied in two clinical studies as a strategy to exploit this receptor as targeted therapy, thereby lowering the risk of adverse effects in extra-hepatic non-ASGPR expressing tissues [208,209]. However, a crucial consideration for galactose-mediated delivery is that ASGPR1 expression exhibits polarity and zonality on the surface of hepatocytes such that it is highly expressed on the basolateral/sinusoidal membrane of hepatocytes and low on the apical hepatocellular membrane (polarity). Similarly, its expression is higher in the hepatocytes in the centrolobular areas as compared to the portalobular regions (zonality) [210]. Several studies have demonstrated the use of nanotechnology in ASGPR targeting in HCC (Table 1).

#### 4.1.2. Glypican-3 (GPC3)

Glypican-3 (GPC3, also known as GTR-2, DGSX, MXR7, OCI-5, SDYS, SGB, SGBS, SGBS1, or heparan sulfate proteoglycan (HSPG)) is a proteoglycan member of the glypican family that is attached to the cell surface by a glycosyl-phosphatidyl-inositol anchor [216,217,218,219]. The GPC3 core protein is a 70 kDa protein with a furin cleavage site located in the middle. This furin cleavage leads to the formation of the 40 kDa N-terminal fragment and the 30 kDa C-terminal fragment [219]. They regulate the signaling activity of several growth factors, including Wnt (Figure 1). Active targeting can overcome barriers to drug delivery by using a moiety that interacts specifically with receptors overexpressed by tumor cells. Studies have shown successful GPC3 targeting for drug delivery to HCC due to its overexpression in the tumor, as opposed to normal and cirrhotic liver, where it is not detected. Previous studies have shown significant success in targeting HCC cells using a humanized anti-GPC3 monoclonal antibody (GC33), and results indicated significant inhibition of GPC3-positive human HCC xenograft tumor growth in SCID mice while the GPC3-negative HCC xenografts were unaffected, proving the potential role of targeting HCC tumor cells through this ligand [216,220]. Table 2; Table 3 demonstrate the different antibodies under research and studies that use nanotechnology in GPC3 targeting in HCC.

#### 4.1.3. Transferrin Receptor (TfR)

TfR is a glycoprotein that plays an important role in iron regulation and cell growth. Iron-bound transferrin has a high affinity for TfR; therefore, the combination of the ligand with the receptor leads to endocytosis through which the iron-bound TfR complex is internalized, releasing the iron and further recycling the receptors back to the surface in an acidic environment [229].

The liver, being the most important organ related to iron storage, is closely linked to iron metabolism and expression of TFR1. Previous literature has shown that iron metabolism is altered in HCC. Significantly higher mRNA levels of genes such as TFR1 participating in the uptake of iron have been detected, making this receptor a potential target for active targeting strategies [230,231,232]. Currently, Tf has been extensively used as a targeting ligand; however, its application is limited due to the presence of high levels of endogenous Tf (25 µM) in human blood (Table 4, Figure 1). The endogenous Tf competitively inhibits the Tf-modified drug delivery systems, which may reduce targeting efficiency in vivo and thereby restrict the application of Tf as a targeting ligand for nanomedicine [233,234,235,236,237,238,239,240,241].

#### 4.1.4. Folic Acid Receptor (FR)

The folic acid receptor, a 38 kDa glycosylphos-phatidylinositol membrane-anchored glycoprotein, is overexpressed in several cancers, including HCC (Figure 1) [251,252,253,254,255]. Reports have shown that FR, especially FR-α expression, is significantly increased in malignant tissues with high affinity toward folic acid as compared to normal liver tissues making it an ideal target for drug delivery to the liver [106,256]. Folic acid has been used over the past several years as an attractive target to deliver cargo to the liver tissue due to its specificity, small size, stability, non-immunogenic and inexpensive properties [257,258]. Mechanistically, folic acid has proven to be vital for fast multiplying cells for DNA synthesis and replication, cell division, growth, and survival, and its deficiency is often associated with abnormal methylation and increased chromosomal strand breaking [259,260,261]. Therefore, taking advantage of the significant role of folic acid in HCC would provide a promising targeting moiety for nanomedicine. Several studies have used folic acid as a targeting agent in HCC, as shown below (Table 5).

#### 4.1.5. Scavenger Receptor Class B Type I (SR-B1)

In addition to various different lipoprotein receptors, SR-B1 is a multiligand membrane receptor protein that possesses ligand properties for cholesterol transport to liver cells (Figure 1). Therefore, SR-B1 plays a central role in cholesterol homeostasis and is a vital element required to maintain cell plasma stability, fluidity, and organization to the liver [275,276,277]. SR-B1 is broadly overexpressed on hepatocytes and responsible for HDL uptake, making HDL mimics a subject of much interest as a promising, versatile, and efficacious target toward HCC [278,279,280,281,282]. HDLs have consistently drawn attention in targeting HCC due to the increased expression of HDL receptors such as SR-B1 in order to satisfy the tumors’ insistent appetite for cholesterol for cell proliferation. Studies have shown HDL targeting using various components of the HDL lifecycle in the liver, whereby LDL is responsible for cholesterol delivery to cells through LDL receptor-mediated endocytosis followed by removal of cholesterol from the periphery and delivering it back to the liver for excretion via HDL [283,284,285]. HDL mimics have shown advantages for nano delivery due to their size and surface properties resembling the native HDL and additionally, protecting them from clearance as compared to other foreign bodies [275,286]. Indeed, with its capacity to provide a gateway for delivering therapeutic agents through HDL mimics such as apoA-1, it has found extensive success in various other cancers and therefore has a potential for delivering nanoparticles as selective and specific HCC therapy (Table 6).

### 4.2. Emerging Use of Peptides in HCC Therapy as an Evolutionary Improvement in HCC Nanomedicine

With the growing research in search of therapeutic options for HCC, studies have identified proteins that are specifically expressed by tumoral cells. These proteins could be used to generate specific peptides to solve the two major obstacles of tumoral specificity and selectivity in HCC targeting [289]. Peptides, by virtue of their small size, possess a number of unique features defining them as promising therapeutic agents that include effective tissue penetration as well as negligence to the host immune system leading to lesser or no off-target effects and adverse effects [290]. Some of the studied peptides in HCC include SP94 [291,292,293], Tv1 [294,295,296,297], FFW [298,299], iRGD [83,91,300], GG-8-6 [301], BR2 [302,303], β3 [304], GW-H1 [305,306], bovicin HC5 [307], R-Tf-D-LP4 [308], C7 [309], HCC79 [289,310], GPC3 peptide [311,312,313] and cecropinXJ [290,314]. Peptides are also being investigated in HCC vaccine development, targeting specifically overexpressed targets such as GPC3 [315]. Following completion of a Phase 1 study of a GPC3 targeting peptide, it was further concluded that the antitumor effects of the peptide alone could not be significant enough, and therefore, the future prospects would be to develop combinational therapies [315]. Therefore, the future lies in using peptides as a targeting modality in drug-encapsulated nanoformulations as a combinational therapy that could make up for the relatively low affinity of peptides and improve antitumor activity.

### 4.3. Nanomedicine as a Vehicle for Delivery of Chemotherapy and siRNA into HCC Cells

Overall, the summary of the nanoparticle literature above provides insight into the variety of intracellular targets that have been targeted with chemotherapeutic agents or siRNA as cargo with nanoparticles as the vehicle for delivery. The nanoparticles facilitate endocytosis of the cargo into the HCC cells. Chemotherapeutic agents encapsulated into nanoparticles have included doxorubicin, paclitaxel, docetaxel, sorafenib, 5-fluoruracil, and gemcitabine. This approach was shown to increase retention time within the tumor cells and effectively inhibit tumor growth in preclinical models of HCC. The single interference RNA approach has also been leveraged in combination with nanoparticles for the treatment of HCC in preclinical models. Some of the targeted genes include survivin, SALL4 and cyclin E [316], NET-1 [317], EMS1 [317], COP9, VEGF [317,318], Bmi1 [140], midkine [319], livin [320,321], c-Myc [322,323], p28GANK [324], CXCR4 [325], DP-1 [134], and integrin B1 [133,326,327,328,329,330,331]. Beyond specific genes, the targeting of a specific miRNA with the siRNA approach has also been evaluated in preclinical models; for example, miRNA-21, miRNA-320a, miRNA-101, and miRNA-122 were preferably taken up by hepatocytes and the tumor cells, further demonstrating a decrease in tumor burden [322,332,333,334].

## 5. Conclusions

The cutting-edge research and development in HCC nanomedicine have provided a powerful tool over traditional approaches for specific tumor targeting. Although designing a nano-drug delivery system is a complex process that requires optimization of its physicochemical properties, targeting HCC cells also requires a thorough understanding of the challenges such as a cirrhotic liver setting and the interaction between nanoparticles and the HCC tumor environment hindering its transition to clinical practice. There are several significant advantages of nanotechnology, ranging from effective targeting to reduced systemic toxicity. Currently, the only FDA-approved nanomedicine for various other cancer treatments includes; Doxil (liposomal doxorubicin), Onivyde (liposomal irinotecan), Abraxane (albumin-particle bound paclitaxel), Eligard (leuprolide acetate), and Vyxeos (liposomal cytarabine and daunorubicin) with none specific to HCC. Interestingly, the most crucial part of designing HCC nanomedicine requires formulating nano systems with ligands specific to the receptors discussed above, such as ASGPR, GPC3, TfR, FR, and SR-B1. Nano systems with such targeting ligands have proven their efficacy for anti-cancer treatment in several in vitro and in vivo studies. Therefore, there has been progressive development in specific targeted nano delivery systems for HCC, and there is excellent potential for translation of this strategy into the clinical context.

## Figures and Tables

**Table 1:** Different nanoformulations showing ASGPR targeting in HCC.

Targeting Moiety	Nanocarrier	Cargo Carried by Nanocarrier	In Vitro and/or In Vivo Studies and Results
Pullulan (Pul), Arabinogalactan (AGn), and Pul-AGn [45]	Polyethylene sebacate (PES) nanoparticles	Doxorubicin	ASGPR-mediated uptake in HepG2 cells, biodistribution and hepatic disposition in vivo and antitumor activity and toxicity testing in vivo showed that Pul and Pul-AGn labeling increased liver uptake with hepatocyte: nonparenchymal cell ratio of 85:15
Lactose [211]	Shell cross-linking nanoparticles	Doxorubicin	In vitro cytotoxicity and cellular uptake in HepG2 cells showed that lactose conjugated NPs were internalized through a lactose-mediated mechanism
Galactose [212]	Cyclodextrins	Not applicable	In vitro and in vivo adherence of hepatocytes to formulation proved that the enzymatically synthesized NPs were specific to hepatocytes
Galactose [213]	Cross-linked pH-sensitive micelles	Paclitaxel	In vitro hepatoma targeting in HepG2 cells and in vivo biodistribution and antitumor studies showed that galactose conjugated NPs underwent receptor-mediated endocytosis mechanism in vitro with enhanced drug accumulation at the tumor sites in vivo
Galactosamine [214]	Albumin nanoparticles	Doxorubicin	In vitro cytotoxicity and cellular uptake in HepG2 concluded that the NPs were selectively taken up by HepG2 cells due to the surface ASGPR as opposed to ASGPR-negative cells
Lactoferrin [215]	PEGylated liposomes	Not applicable	In vitro cellular uptake in HepG2 lines and in vivo imaging for targeting in HepG2 bearing mice showed that cell uptake was efficiently associated with ASGPR-positive HepG2 cells compared to negative control along with increased drug accumulation in tumors treated with conjugated NPs

**Table 2:** Summary of the various GPC3-targeting antibodies currently under research.

Antibody	Species	Antibody Form	Mechanism of Action	Development Status
GC33 [217,220,221,222,223,224]	Humanized mouse	IgG	Antibody-dependent cellular cytotoxicity (ADCC)	Phase II
YP7 [217,225]	Humanized mouse	IgG	ADCC	Preclinical
HN3 [217,226]	Human	VH-hFc	Inhibition of YAP signaling, direct inhibition of HCC cell proliferation	Preclinical
MDX-1414 [217]	Human	IgG	Not available	Preclinical

**Table 3:** Summary of different nanoformulations designed using GPC3 as a targeting ligand.

Targeting Moiety	Nanocarrier	Cargo Carried by Nanocarrier	In Vitro and/or In Vivo Studies and Results
Peptides specific for GPC3 [97]	Lipid nanoparticles	Sorafenib	In vitro cytotoxicity, cellular uptake in Hep3B and SK-Hep1 cells, and in vivo targeting and antitumor studies in Hep3B xenografts showed that the effective aqueous solubility of sorafenib was improved with increased uptake in vivo
GPC3 mAb [227]	Iron oxide nanoparticles	N/A	In vitro cell uptake and MRI in HepG2 and HLF lines confirmed that only GPC3-expressing cells were specifically targeted using these NPs and may increase pre-treatment MR imaging capability for HCC visualization
GPC3 mAb [228]	Citrate-coated nanoparticles	Prussian blue	In vitro cellular uptake and targeted MR imaging in HepG2 cells confirmed mAb targeting cells via receptor-mediated endocytosis with excellent MR imaging contrast enhancement ability and biocompatibility

**Table 4:** Different nanoformulations using transferrin receptors as a potential HCC target.

Targeting Moiety	Nanocarrier	Cargo Carried by Nanocarrier	In Vitro and/or In Vivo Studies and Results
Transferrin [242]	PVA and albumin nanoshells	Doxorubicin and sorafenib	In vitro TFR1 expression, cytotoxicity, apoptosis, cellular uptake in HepG2 cells and 3D HCC spheroids showed synergistic cellular uptake and cytotoxicity in 92% of cells with efficient cell penetration
Transferrin [243]	Sodium hexametaphosphate gold nanoparticles	N/A	In vitro cytotoxicity and cellular uptake in J5 cells showed its biocompatibility along with higher cellular intake compared to non-conjugated NPs
Apotransferrin Lactoferrin [244]	Lipid nanoparticles	Doxorubicin	In vivo anti-cancer effect in diethylnitrosamine (DENA)-induced HCC rats confirmed significant antitumor potential by reduced liver nodules along with extended bioavailability of the designed NPs
TfR-specific peptide (HAIYPRH) [245]	Polyamidoamine dendrimer	pORF-hTRAIL and doxorubicin	In vitro cellular uptake and gene expression studies in Bel-7402 cells and in vivo antitumor and apoptotic effect in Bel-7402-derived xenografts showed increased cellular uptake and induction of apoptosis. In vivo study showed efficient tumor accumulation of NPs
Apotransferrin [246]	Inorganic nanoparticles	Cisplatin	In vitro cytotoxicity, cellular uptake, and pathway analysis in HepG2 cells showed specific targeting of NPs supported by apoptosis of HepG2 cells induced by the conjugate
Transferrin [247]	Liposomes	DNA	In vitro cellular uptake and gene expression in Huh7 and SK-Hep1 lines followed by in vivo gene transfer efficiency using VX2 rabbit hepatocarcinoma model depicted increased dose-dependent targeting using transferrin
Transferrin [248]	Lipid nanoparticles	N/A	In vitro cytotoxicity in HepG2 and KC cells and in vivo transfection activity in HepG2 xenografts concluded high loading efficiency along with excellent cell targeting ability of Tf-conjugated NPs
Transferrin [249]	Quantum dots (nanocrystales)	N/A	In vitro receptor activation analysis in HepG2 cells demonstrated that the Tf-conjugated quantum dots (QDs) were internalized and tightly bound to the cell receptors conferring it a useful technique in biological labeling of cells
Transferrin [250]	Liposomes	DNA	In vivo gene delivery analysis in VX2 hepatocarcinoma rabbit model demonstrated NPs accumulated only in tumor cells using transarterial injection compared to intra-tumoral injection that transfected peritumoral cells along with hepatic tumor cells
LT7 (L(HAIYPRH)) [233]	Liposomes	N/A	In vitro binding affinity and cellular uptake in HepG2 cells followed by in vivo antitumor effect in HepG2-derived xenografts showed better cellular uptake and enhanced antitumor effect using NPs along with enhanced drug accumulation in HCC

**Table 5:** Summary of various nanoformulations using folic acid as a potential target to deliver drugs to HCC tumor cells.

Targeting Moiety	Nanocarrier	Cargo Carried by Nanocarrier	In Vitro and/or In Vivo Studies and Results
Folic acid [262]	Pluronic F127 nanomicelles	Silibinin	In vitro cytotoxicity in HepG2 cells indicated that the viability of cells treated with conjugated NPs was significantly less than unconjugated NPs
Folic acid [263]	Mn-ZnS quantum dots with chitosan biopolymer	5-Fluorouracil	In vitro drug release and in vivo sub-chronic toxicity assay and anti-4T1 breast cancer study indicated a controlled release behavior in vitro and accumulation of NPs in the tumor of the tumor-bearing mice
Folic acid [264]	Casein micelles	Berberine and diosmin	In vivo cytotoxicity and uptake studies using HepG2 cells supported with in vivo antitumor efficacy using DENA-induced HCC mouse model demonstrated superior cytotoxicity and cellular uptake in vitro along with increased antitumor efficacy in vivo
Folic acid and/or bevacizumab (dual targeting) [265]	Carbon dots	Gadolinium (imaging nanoprobe)	In vitro cytotoxicity assay, fluorescent imaging, and cellular uptake using Hepa1-6 and L929 cells indicated low toxicity with improved sensitivity and specificity as an ideal fluorophore nanosystem
Folic acid [266]	Human serum albumin nanoparticles	Sorafenib	In vitro cell viability assay, cellular uptake, and apoptosis analysis using BEL-7402 cells along with in vivo antitumor efficacy and safety evaluation and tissue distribution study using BEL-7402 xenograft model and pharmacokinetic study confirmed enhanced cytotoxicity, increased safety, and notably enhanced sorafenib accumulation in tumor tissues in vivo
Folic acid [267]	Quantum dots	5-fluorouracil	In vitro cellular uptake using HepG2 cells and in vivo antitumor efficacy, toxicity, and biodistribution study using SMMC-7721 xenograft model indicated reduced cytotoxicity compared to free drug in vitro and enhanced tumor suppression in vivo
Folic acid [268]	ZIF-8 nanoparticles	Doxorubicin	In vitro cytotoxicity using HepG2 cells showed higher anti-cancer efficiency as a targeted therapy
Folic acid and Transferrin [269]	Graphene oxide DDS	Doxorubicin	In vitro cytotoxicity using SMMC-7721 cell line indicated that the double target drug delivery system exhibited controlled drug release, no toxicity, and better inhibitory effect on HCC cells
Adamantanyl-folic acid [270]	Ternary nanoassembly	Pheophorbide	In vitro cellular uptake, photodynamic therapy, and apoptosis assay in MCF-7 and PC3 cells demonstrated increased cellular uptake and improved phototoxicity
PEG-Folic acid [271]	Gold nanocages	Anti-miR-181b	In vitro cellular uptake and cytotoxicity assay using SMMC-7721 cells accompanied with in vivo biodistribution and antitumor effect in SMMC-7721 xenograft model indicated efficient cargo delivery in vitro and suppressing tumor growth and significantly reducing tumor volumes in vivo
Folic acid [272]	Liposomes	HSV-TK suicide gene	In vitro cellular uptake and cell viability using SMMC-7721, HepG2, and HL-7702 cells and in vivo tumor and tissue distribution imaging using Bel-7402 xenograft model and antitumor efficacy using Bel-7402 xenograft model demonstrated highly tumor-specific imaging and excellent antitumor activity devoid of any systemic toxicity
Folic acid [106]	Selenium nanoparticles	HES5 siRNA	In vitro cellular uptake, cytotoxicity, and apoptosis assay using HepG2 and Lo2 cell lines along with in vivo biodistribution and antitumor efficacy using HepG2 xenograft model indicated higher cellular uptake, enhanced gene silencing efficiency, and increased cytotoxicity in vitro. NPs conjugated with FA showed increased antitumor efficacy and low toxicity compared to unconjugated NPs
Folic acid [256]	Drug delivery vehicle	Fluorescein	In vitro cytotoxicity and apoptosis assay using N1S1 and U937 cell lines supported with in vivo biodistribution using N1S1 cell-induced murine model. Results indicated increased specific uptake due to cell surface folate receptors
Folic acid [273]	Porphyrin nanoparticles	Gadolinium	In vitro biotoxicity and imaging capability using HepG2 cells and in vivo biotoxicity and imaging capability in embryonic and larval zebrafish demonstrated excellent MRI capability both in vitro and in vivo due to its strong affinity for folate receptor on HCC tumor cells
Folic acid [274]	pH-sensitive nanoparticles	Triptolide	In vitro cytotoxicity and cellular uptake using Bel-7404 cells and in vivo distribution and antitumor effect using Bel-7404 xenograft model indicated that the pH-sensitive NPs increased cellular uptake mitigating the significant toxicity of triptolide

**Table 6:** Brief summary showing the different nanoformulations designed using SR-B1 receptor as a target.

Targeting Moiety	Nanocarrier	Cargo Carried by Nanocarrier	In Vitro and/or In Vivo Studies and Results
ApoA-1 [287]	Lipid nanoparticles	SALL4 siRNA	In vitro cellular uptake assay and silencing efficacy in KB, HT1080, Hep3B, SNU398, and Huh7 cell lines. Along with in vivo biodistribution and antitumor effect in KB, HT1080, and Hep3B xenograft model. Results showed inhibited tumor growth with a 3-fold increase in apoptosis
ApoA-1 [288]	Lipid nanoparticles	Doxorubicin	In vitro cytotoxicity and cellular uptake assay in HepG2 cells indicated increased cytotoxicity and cellular uptake in SR-B1-positive liver cells
ApoA-1 [286]	Lipid nanoparticles	Vadimezan and gemcitabine	In vivo imaging, biodistribution, and antitumor effect in Hepa1-6 xenograft model demonstrated specific targeting to HCC tumors
